# Supplementary material for: Reconfigurable exciton-plasmon interconversion for nanophotonic circuits
Source: Nat Commun. 2016 Nov 28;7:13663. doi: 10.1038/ncomms13663 (PMC5133701; doi:10.1038/ncomms13663)
Supplement: Supplementary Information — Supplementary Figures 1-8, Supplementary Notes 1-8 and Supplementary References. [file ncomms13663-s1.pdf]

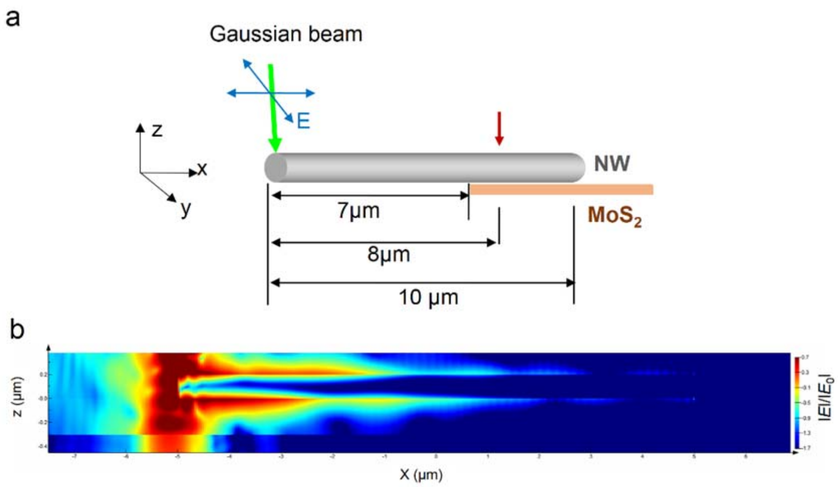

Supplementary Figure 1 | FDTD simulation for Ag-NW/MoS_2 hybrids. (a) Schematic of the FDTD numerical simulation for excitonic transistors. **(b)** The cross section of the optical field map in the NW axial direction (x-axis).

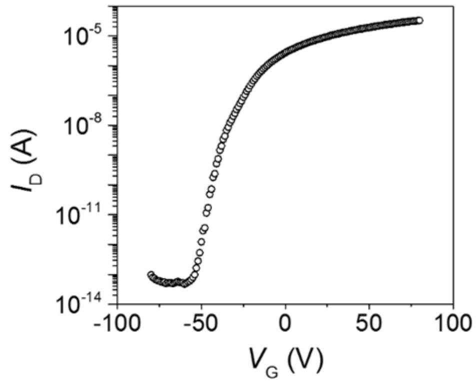

Supplementary Figure 2 | I_D-V_G transfer curve for MoS_2 FETs. Log-scale drain current (I_D) characteristics at a drain bias (V_D) of 1 V as a function of gate bias (V_G) for MoS_2 FETs.

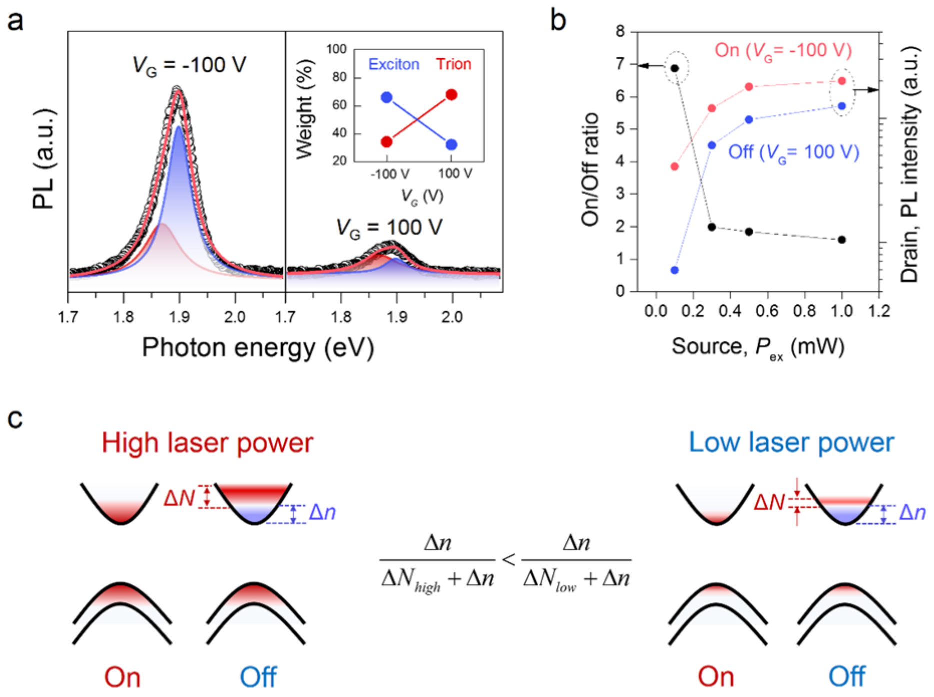

Supplementary Figure 3 | PL spectra for On- and Off-V_G and P_{ex} dependence of On/Off ratio in the Ag-NW/MoS_2 hybrid. (a) PL deconvolution by excitons and trions for On (V_G = −100 V) and Off (V_G = 100 V) states at P_{ex}= 0.1 mW for Fig. 1d. Inset: exciton and trion spectral weights for On and Off states. **(b)** Integrated PL intensity for On (V_G = −100 V) and Off (V_G = 100 V) states and On/Off ratio at D as a function of P_{ex}. **(c)** The schematic of the exciton switching efficiency via the modulation of electrical doping, Δn, for high (ΔN_{high}) and low (ΔN_{low}) laser powers. ΔN: the photocarrier density of MoS_2.

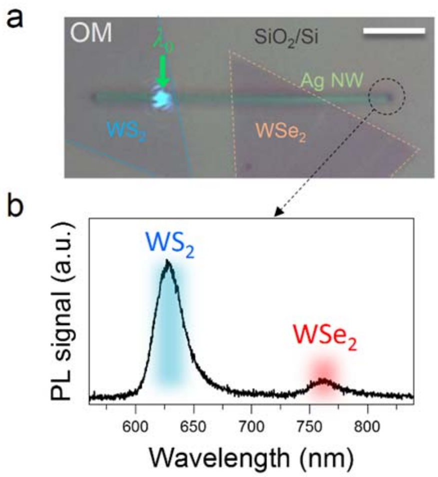

Supplementary Figure 4 | Exciton multiplexing in the Ag-NW interconnected WS_2-WSe_2 hybrid. (a) Optical micrograph (OM) overlapped with the device structure. WSe_2 and WS_2 monolayers on SiO_2 (300 nm)/Si wafers are bridged by the Ag-NW. The green arrow is the input laser position, the black dashed circle is the PL collection position at λ_0 = 514 nm. Scale bar: 5 μm. **(b)** PL spectrum measurd at the black dashed circle in **(a)**.

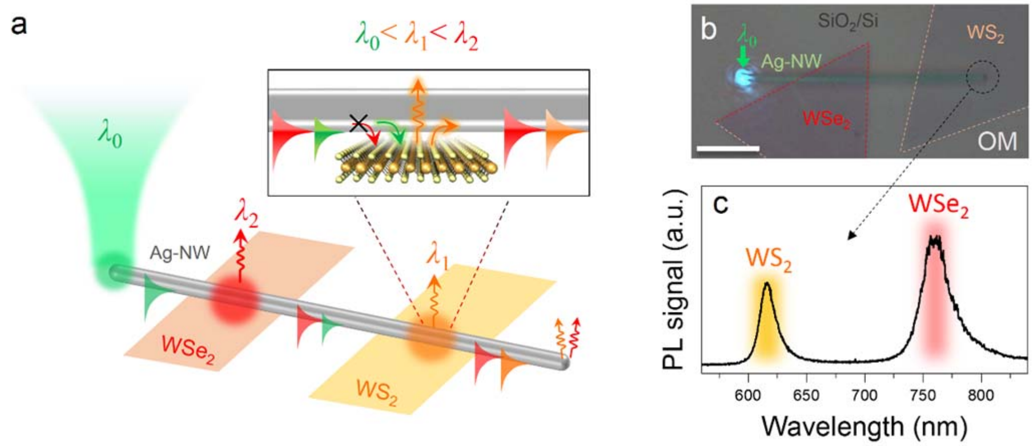

Supplementary Figure 5 | Exciton multiplexing without the re-absorption interaction in the Ag-NW interconnected WS_2-WSe_2 hybrid. (a) Schematic of the device configuration. The WSe_2 and WS_2 monolayers are bridged by the Ag-NW. During illumination of the focused laser light (λ_0 = 514 nm, green color) at the left end of the NW, generated λ_0-coupled SPPs propagate along the NW and excite the WSe_2 excitons (λ_2, red color). Both λ_0- and λ_2-coupled SPPs propagate along the NW and arrive at the WS_2 layer. λ_0-coupled SPPs are absorbed in WS_2 and generate the WS_2 excitons (λ_1, orange color), and λ_2-coupled SPPs transmit without absorption. Individually multiplexed λ_1- and λ_2-coupled SPPs de-multiplex at the NW end. **(b)** Optical micrograph (OM) overlapped with the schematic of the TMD flakes and the NW. The green arrow is the input laser position and the black dashed circle is the PL collection position. Scale bar: 5 μm. **(c)** PL spectrum measured at the black dashed circle in (**b**).

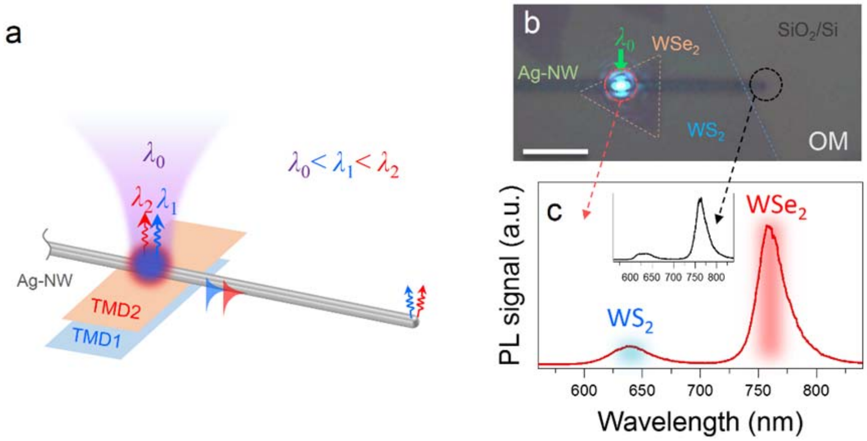

Supplementary Figure 6 | Exciton multiplexing in the Ag-NW overlapping on the vertically stacked WSe_2/WS_2 hybrid. (a) The Ag-NW is partially overlapped on the sequentially stacked WSe_2/WS_2 heterojunction. The excitons of WS_2 (λ_1, blue color) and WSe_2 (λ_2, red color) are simultaneously generated by λ_0 and coupled to the SPPs. **(b)** Optical micrograph (OM) overlapped with the device structure. The green arrow is the input laser position, the red and black dashed circles are the PL collection positions. Scale bar: 5 µm. **(c)** PL spectrum measured at the NW mid-section (red dashed circle). The inset: the PL spectrum measured at the NW end (black dashed circle).

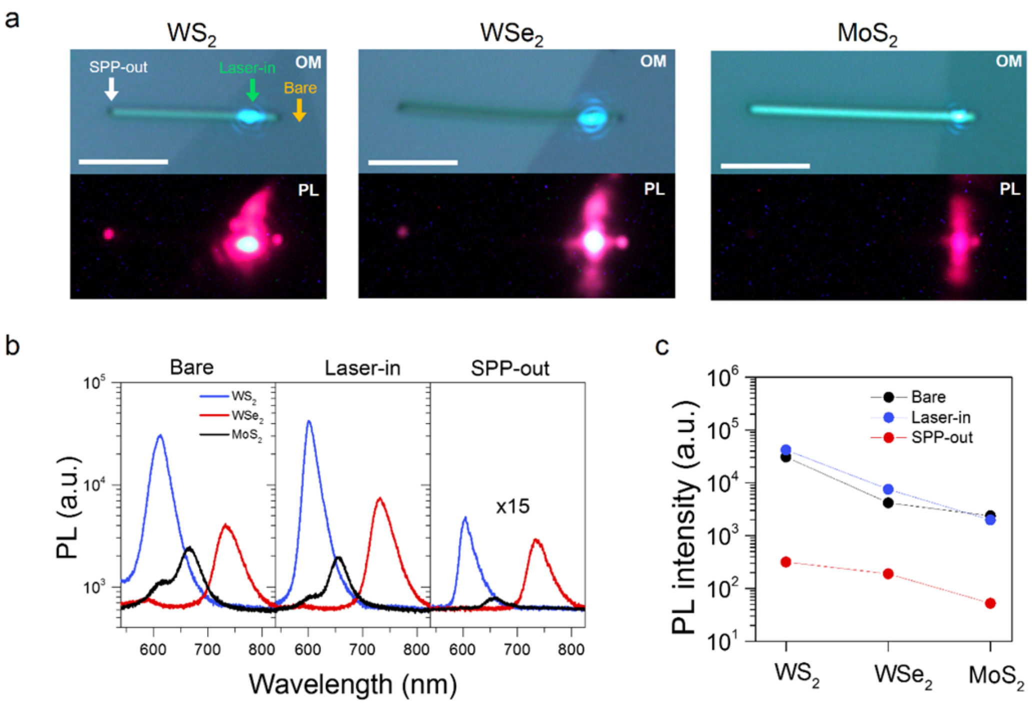

Supplementary Figure 7 | Exciton-plasmon interconversion for various TMD materials. **(a)** Optical micrographs under a laser illumination and their PL images for three different NW/TMD hybrids. The Ag-NW is partially overlapped on each WS_2 (left), WSe_2 (mid), and MoS_2 (right), respectively. Green arrow: laser input position. Whit arrow: NW end position. Orange arrow: bare TMD position. Scale bar: 5 μm. **(b)** PL spectra collected at the three different samples. **(c)** Comparative maximum PL intensities obtained from the PL spectra **(b)** for each TMD.

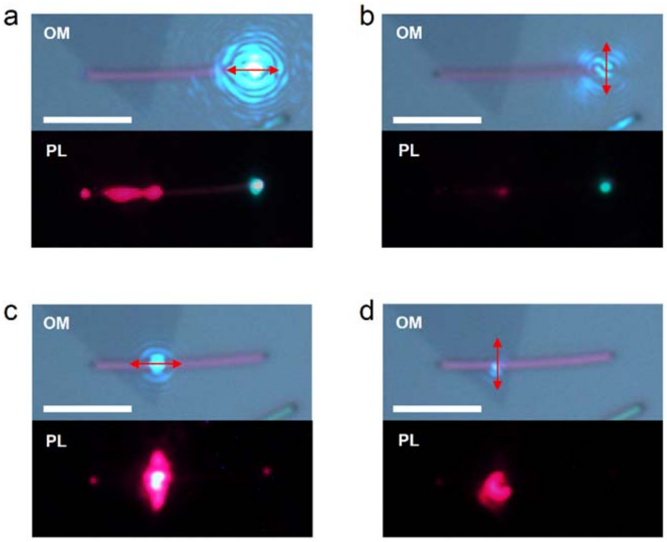

Supplementary Figure 8 | Polarization effect in exciton-plasmon interconversions. The Ag-NW is partially overlapped on WS_2. The polarized laser beams are illuminated at the NW end **(a and c)** and the NW/WS_2 overlapping region **(b and d)**. Red arrow: parallel **(a and c)** and perpendicular **(b and d)** polarizations to the NW direction. Top panel: optical micrograph (OM). Bottom panel: PL image. Scale bar: 5 μm.

Supplementary Note 1: FDTD simulations and SPP propagation along NW channels

A finite-difference time-domain numerical simulation (Lumerical Solutions, Inc.) was conducted for a NW-MoS_2 hybrid on a SiO_2 (300 nm)/Si substrate, as shown in the Fig. 1b. The design for the numerical simulation of the device configuration is illustrated in Supplementary Fig. 1a, where the diameter and length of the Ag-NW were 200 nm and 10 μm, respectively, and the thickness of the MoS_2 monolayer was 0.7 nm. The minimum mesh size near the MoS_2 layer was 0.1 nm. A Gaussian beam (wavelength: 514 nm, beam radius: 180 nm) was used as the input laser source[1]. The cross-sectional optical field for Fig. 1b is monitored at the position indicated by a red arrow in Supplementary Fig. 1a. Supplementary Figure 1b shows the cross-sectional optical field (log scale) in the axial direction. Confined optical fields near the NW propagate along the NW and arrive at the MoS_2 region, as demonstrated in the experiment (Fig. 1).

The SPPs propagate along the NW channel with a tight confinement of optical mode near the NWs, where an effective mode area (A_m) for the NW waveguides is given by[2]

$$A_m = \frac{W_m}{\max\{W(\mathrm{r})\}}, \quad (1)$$

where $W_m = \int_{A_m} W(\mathrm{r})dA$ is the total mode energy and $W(\mathrm{r})$ is the energy density per unit length along the propagation direction. For the complex dielectric function of a material, $\varepsilon(\mathrm{r})$, the energy density $W(\mathrm{r})$ at the given frequency ω is expressed as

$$W(\mathrm{r}) = \frac{1}{2}\left\{ \frac{d(\varepsilon(\mathrm{r})\omega)}{d\omega} |E(\mathrm{r})|^2 + \mu_0 |H(\mathrm{r})|^2 \right\}. \quad (2)$$

The SPP propagation length for $\hbar\omega_0$ is $L_0 = 1/\{2\,\mathrm{Im}[k_{SPP}(\omega_0)]\}$, where $k_{SPP}(\omega_0)$ is the complex wavevector of the SPP mode in the propagation direction[2].

Supplementary Note 2: Fermi energy level derived from electrostatic doping

Supplementary Figure 2 shows a transient drain current (I_D) versus V_G at a drain bias (V_D) of 1 V at room temperature. Transient position of I_D is near −50 V so that the charge neutrality point is taken to V_G =−50 V. From the back-gate capacitance, the carrier densities for electrons and holes were calculated using the relation $ne = C_i V_G$, where $C_i = \varepsilon_0 \varepsilon_r / d = 11.5\times10^{-9}$ F/ cm^2, $\varepsilon_r = 3.9$, $d =$ 300 nm, and n and e denote the carrier density and electron charge, respectively[3]. The effective masses of electrons and holes for MoS_2 monolayers are given by $m_e = 0.35 m_0$, $m_h = 0.44 m_0$, respectively, where m_0 denotes electron mass[4] . Using the relation $\mu_e = \hbar^2 \pi n / m_e$ for electrons and $\mu_h = \hbar^2 \pi n / m_h$ for holes, the Fermi level was calculated[5] as shown in Fig. 1e.

Supplementary Note 3: V_G-doping and SPP intensity effect in exciton flux modulations

Figure 1d shows the V_G-dependent PL spectra measured in the drain region of the Ag-NW/MoS_2 FET hybrid. With increasing electrical doping (V_G < 0 V), the PL intensities gradually decrease but the peak position shift is not prominent. Supplementary Figure 3a shows the PL spectra for the On (V_G = −100 V) and Off (V_G = 100 V) states deconvoluted by the Lorentzian function for the exciton and trion. Here, the peak positions for the exciton and trion are assigned to 1.9 eV and 1.87 eV due to the trion binding energy of ~30 meV[6]. With increasing electron doping for the On state, the PL intensity decreases due to Pauli blocking effect[5,6] but the negative trion population ratio increases (Supplementary Fig. 3a, inset), obeying the mass action law for trions[7].

The intensity of the photon-coupled-SPPs is proportional to the λ_0 illumination intensity. To investigate the SPP intensity effect in the exciton flux modulation, the integrated PL intensities for On state (V_G = −100 V) and Off state (V_G = 100 V) as functions of the input laser power (P_{ex}) were measured at the NW/ MoS_2 overlapping region (Supplementary Fig. 3b, right axis) and their On/Off ratio was displayed in left axis in Supplementary Fig. 3b as discussed in the Fig. 1f. As P_{ex} is reduced, On/Off ratio increases and reaches a maximum value of ~7 at P_{ex} = 0.1 mW and the PL intensities for both states decrease. At high P_{ex}, the photocarrier density (ΔN) of MoS_2 generated by the SPPs is higher than that at a low P_{ex}. Therefore, $\Delta N_{high} > \Delta N_{low}$. The exciton switching efficiency due to the electrical doping effects for the blocking exciton generation is proportional to $\Delta n / (\Delta N + \Delta n)$; thus, $\Delta n / (\Delta N_{high} + \Delta n) < \Delta n / (\Delta N_{low} + \Delta n)$. Accordingly, at a low P_{ex}, the exciton switching efficiency is more effective than that at a high P_{ex}, as schematically depicted in Supplementary Fig. 3c. The P_{ex} dependence in the flux modulation agrees well with MoS_2 monolayers[6].

Supplementary Note 4: Exciton multiplexing in WS_2-NW-WSe_2 hybrids

The concept of exciton multiplexing is demonstrated in a WS_2-NW-WSe_2 hybrid (Supplementary Fig. 4). As shown in a representative optical micrograph (Supplementary Fig. 4a), the WS_2 and WSe_2 monolayers are interconnected by an Ag-NW. In the NW/WS_2 overlapping region, the focused laser light (λ_0 = 514 nm) is illuminated, which is indicated by a green arrow (Supplementary Fig. 4a). At the same time, the photoluminescence (PL) signal is measured at the NW end (black dashed circle). The WS_2 excitons excited by input light (λ_0) are coupled to WS_2-exciton-coupled-SPPs in the NW. The SPPs propagate along the NW and are absorbed by the WSe_2 layer. As a result, the WSe_2 excitons excited by the WS_2-exciton-coupled-SPPs are coupled to WSe_2-exciton-coupled-SPPs in the NW. Eventually, WS_2- and WSe_2-exciton-coupled-SPPs are multiplexed in the single NW and the de-multiplexed spectra are monitored at the end of the NW owing to the radiative emission of the SPP modes[1,8]. Supplementary Figure 4b shows the measured PL spectrum. The de-multiplexed exciton signals for WS_2 and WSe_2 are observed near wavelengths of 620 and 760 nm, respectively.

Supplementary Note 5: Multiplexing strategy for individual wavelength modulations

Supplementary Figure 5a illustrates a multiplexing method for individual wavelength modulation. The WS_2 ($\lambda_1 \approx$ 620nm) and WSe_2 ($\lambda_2 \approx$ 760 nm) monolayers on the SiO_2 (300 nm)/Si wafers are interconnected by the Ag-NW. The excitation light (λ_0 = 514 nm) is illuminated at the left end of the NW and λ_0-coupled SPPs propagate along the NW, exciting λ_2 and λ_1 sequentially. Here, λ_2-coupled SPPs transmit without absorption in WS_2 due to λ_1 being less than λ_2 and arrive at the right end of the NW. To demonstrate this concept, the sample shown in the optical micrograph of Supplementary Figure 5b. At the right end of the NW (black dashed circle), the PL spectrum was collected, as shown in Supplementary Figure 5c. The PL intensity of WSe_2 is still higher than that of WS_2 because λ_2-coupled SPPs are not absorbed in the WS_2 layer. This result implies that electrically modulated signals from each TMD can independently generate and the signals deliver when dielectric spacers between the TMDs and the NWs electrically isolate each TMD. Here, λ_2 and λ_1 are converted from λ_0 at each TMD positions.

Supplementary Note 6: Van der Waals stacking for multiple-exciton generation

Our method for multiplexing wavelengths is flexible in the way for reconfiguring devices. Supplementary Figure 6a illustrates the architectural concept of generating the multiple excitonic wavelengths via the van der Waals heterostacking of multilayered tandem structures[9,10]. The WS_2 ($\lambda_1 \approx 620$ nm)[11] and WSe_2 ($\lambda_2 \approx 760$ nm) monolayers are sequentially stacked on the SiO_2 (300 nm)/Si wafers, and the Ag-NW overlaps the TMD bilayers. The excitation light ($\lambda_0 = 514$ nm) is illuminated on a NW/TMD overlapping region and generated λ_1 and λ_2 excitons are simultaneously converted to λ_1- and λ_2-coupled-SPPs, respectively. As shown in a representative optical micrograph (Supplementary Fig. 6b), under the λ_0 (green arrow) illumination on the NW/TMD overlapping region, the PL signal is measured at the same position (red dashed circle) and the right end of the NW (black dashed circle). Supplementary Figure 6c shows the measured PL spectrum. The excitonic signals of WS_2 and WSe_2 are observed near wavelengths of 620 and 760 nm, respectively, and the similar PL spectrum is observed at the right end of the NW (Supplementary Fig. 6c, inset). The result implies that heterojunction multilayers fabricated by van der Waals stacking could be used for generating multiple-exciton sources. Therefore, in our device design, various combinations of wavelengths are available by controlling not only the lateral order of the TMD array but also the vertical heterojunction stacking of the TMD layers.

Supplementary Note 7: Material dependence of the exciton-to-plasmon coupling

We compared the exciton-to-plasmon coupling effect for three different materials. Supplementary Figure 7a shows optical micrographs under a laser illumination and their PL images for the Ag-NW overlapped WS_2 (left), WSe_2 (mid), and MoS_2 (right). Under laser illumination at the NW/TMD overlapped regions, PL signals were collected at the input position of laser (Laser-in, green arrow) and the position of NW end (SPP-out, white arrow) for each sample. For comparison, PL spectra were also measured at the bare TMD regions (Bare, orange arrow). The measured spectra are displayed in Supplementary Fig. 7b. The PL intensity trend (WS_2 > WSe_2 > MoS_2) and peak shape are similar for the three different positions of the sample. Supplementary Figure 7c shows comparative maximum PL intensity curves obtained from the PL spectra of Supplementary Fig. 7b. The PL intensity curves for the Bare and Laser-in are in the similar values. While the PL intensity values for SPP-out are prominently lower than those for the Bare and Laser-in due to the coupling and SPP propagation losses, all curves show similar intensity trend of WS_2 > WSe_2 > MoS_2.

For each sample, the SPP losses are estimated to be the similar order of magnitude (~55% to ~65%) via FDTD mode analyses: the SPP propagating length (from laser input to NW end) for each sample ranging from ~8 μm to ~ 12 μm, the SPP losses for each excitonic wavelength ranging from 600 nm ~ 750 nm. Because of this we assumed that the deviation of SPP propagation losses for each sample is negligible. We conclude that the material dependence of exciton-to-plasmon coupling effects is negligible although the quantum efficiencies of each TMD are prominently different, since the PL intensity of the SPP-out is quantitatively proportional to that of the Laser-in without material dependence (Supplementary Fig. 7c).

Supplementary Note 8: Polarization effect in the exciton-plasmon interconversions

We investigate the polarization effect of light illumination in the exciton-plasmon interconversion using a partially overlapped Ag-NW on WS_2 flake. Supplementary Figure 8a and b show optical micrograph and PL images under laser illumination at the NW end for parallel (Supplementary Fig. 8a) and perpendicular (Supplementary Fig. 8b) polarizations to the NW direction. The laser light coupled SPP propagates along the NW and excites excitons at the NW/WS_2 overlapping region. While a strong red color emission at the NW/WS_2 overlapping region is observed for the parallel polarization (Supplementary Fig. 8a, bottom), the emission is negligible for the perpendicular polarization (Supplementary Fig. 8b, bottom). These results are consistent with the polarization effects in Ag-NWs in previous reports[12].

Supplementary Figure 8 c and d show optical micrograph and PL images under laser illumination at the NW/WS_2 overlapping region for parallel (Supplementary Fig. 8c) and perpendicular (Supplementary Fig. 8d) polarizations to the NW direction. Excited WS_2 excitons at the NW/WS_2 overlapping region propagate along the NW and scattered out at the NW ends. While a strong red color emission at each NW end is observed for the parallel polarization (Supplementary Fig. 8c, bottom), the emission is negligible for the perpendicular polarization (Supplementary Fig. 8d, bottom). The results are consistent with Supplementary Fig. 8 a and b.

Supplementary References

1. Lee, H.S. *et al.* Efficient Exciton-Plasmon Conversion in Ag Nanowire/Monolayer MoS_2 Hybrids: Direct Imaging and Quantitative Estimation of Plasmon Coupling and Propagation. *Adv. Opt. Mater.* **3,** 943 (2015).
2. Guo, X., Ma, Y.G., Wang, Y.P. & Tong, L.M. Nanowire plasmonic waveguides, circuits and devices. *Laser Photonics Rev.* **7,** 855 (2013).
3. Pradhan, N.R. *et al.* Intrinsic carrier mobility of multi-layered MoS_2 field-effect transistors on SiO_2. *Appl. Phys. Lett.* **102,** 123105 (2013).
4. Cheiwchanchamnangij, T. & Lambrecht, W.R.L. Quasiparticle band structure calculation of monolayer, bilayer, and bulk MoS2. *Phys. Rev. B* **85,** 205302 (2012).
5. Newaz, A.K.M. *et al.* Electrical control of optical properties of monolayer MoS_2. *Solid State Commun.* **155,** 49 (2013).
6. Lee, H.S., Kim, M.S., Kim, H. & Lee, Y.H. Identifying multiexcitons in MoS_2 monolayers at room temperature. *Phys. Rev. B* **93,** 140409(R) (2016).
7. Mouri, S., Miyauchi, Y. & Matsuda, K. Tunable Photoluminescence of Monolayer MoS_2 via Chemical Doping. *Nano Lett.* **13,** 5944 (2013).
8. Lee, H.S. *et al.* Selective Amplification of the Primary Exciton in a MoS_2 Monolayer. *Phys. Rev. Lett.* **115,** 226801 (2015).
9. Lee, C.H. *et al.* Atomically thin p-n junctions with van der Waals heterointerfaces. *Nat. Nanotechnol.* **9,** 676 (2014).
10. Gong, Y.J. *et al.* Vertical and in-plane heterostructures from WS_2/MoS_2 monolayers. *Nat. Mater.* **13,** 1135 (2014).
11. Shang, J.Z. *et al.* Observation of Excitonic Fine Structure in a 2D Transition-Metal Dichalcogenide Semiconductor. *ACS Nano* **9,** 647 (2015).
12. Huang, Y.Z., Fang, Y.R., Zhang, Z.L., Zhu, L. & Sun, M.T. Nanowire-supported plasmonic waveguide for remote excitation of surface-enhanced Raman scattering. *Light-Sci. Appl.* **3,** e199 (2014).
